# Evolution and Prognostic Variables of Cystic Fibrosis in Children and Young Adults: A Narrative Review

**DOI:** 10.3390/diagnostics15151940

**Published:** 2025-08-02

**Authors:** Mădălina Andreea Donos, Elena Țarcă, Elena Cojocaru, Viorel Țarcă, Lăcrămioara Ionela Butnariu, Valentin Bernic, Paula Popovici, Solange Tamara Roșu, Mihaela Camelia Tîrnovanu, Nicolae Sebastian Ionescu, Laura Mihaela Trandafir

**Affiliations:** 1 Pediatrics Department, “Grigore T. Popa” University of Medicine and Pharmacy, 700115 Iasi, Romania; madalina.donos@umfiasi.ro (M.A.D.); paula.popovici@umfiasi.ro (P.P.); laura.trandafir@umfiasi.ro (L.M.T.); 2 Department of Surgery II, Pediatric and Orthopedic Surgery, “Grigore T. Popa” University of Medicine and Pharmacy, 700115 Iasi, Romania; 3 Department of Morphofunctional Sciences I—Pathology, “Grigore T. Popa” University of Medicine and Pharmacy, 700115 Iasi, Romania; elena2.cojocaru@umfiasi.ro; 4 Faculty of Medicine, Apollonia University, Strada Păcurari nr. 11, 700511 Iasi, Romania; 5 Department of Medical Genetics, Faculty of Medicine, “Grigore T. Popa” University of Medicine and Pharmacy, 700115 Iasi, Romania; ionela.butnariu@umfiasi.ro; 6 Department of Surgery I, “Grigore T. Popa” University of Medicine and Pharmacy, 700115 Iasi, Romania; bernicvalik@yahoo.com; 7 Department of Nursing, Faculty of Medicine, “Grigore T. Popa” University of Medicine and Pharmacy, 700115 Iasi, Romania; rosusolange@yahoo.com; 8 Department of Mother and Child Medicine, “Grigore. T. Popa” University of Medicine and Pharmacy, 700115 Iasi, Romania; mihaela.tirnovanu@umfiasi.ro; 9 Department of Pediatric Surgery and Orthopedics, “Carol Davila” University of Medicine and Pharmacy, 020021 Bucharest, Romania; sebastian.ionescu@umfcd.ro; 10 Romanian Academy of Medical Sciences, 030167 Bucharest, Romania

**Keywords:** cystic fibrosis, diagnostic, management, mucoviscidosis

## Abstract

**Introduction:** Cystic fibrosis (CF) is a genetic condition affecting several organs and systems, including the pancreas, colon, respiratory system, and reproductive system. The detection of a growing number of *CFTR* variants and genotypes has contributed to an increase in the CF population which, in turn, has had an impact on the overall statistics regarding the prognosis and outcome of the condition. Given the increase in life expectancy, it is critical to better predict outcomes and prognosticate in CF. Thus, each person’s choice to aggressively treat specific disease components can be more appropriate and tailored, further increasing survival. **The objective** of our narrative review is to summarize the most recent information concerning the value and significance of clinical parameters in predicting outcomes, such as gender, diabetes, liver and pancreatic status, lung function, radiography, bacteriology, and blood and sputum biomarkers of inflammation and disease, and how variations in these parameters affect prognosis from the prenatal stage to maturity. **Materials and methods**: A methodological search of the available data was performed with regard to prognostic factors in the evolution of CF in children and young adults. We evaluated articles from the PubMed academic search engine using the following search terms: prognostic factors AND children AND cystic fibrosis OR mucoviscidosis. **Results:** We found that it is crucial to customize CF patients’ care based on their unique clinical and biological parameters, genetics, and related comorbidities. **Conclusions**: The predictive significance of more dynamic clinical condition markers provides more realistic future objectives to center treatment and targets for each patient. Over the past ten years, improvements in care, diagnostics, and treatment have impacted the prognosis for CF. Although genotyping offers a way to categorize CF to direct research and treatment, it is crucial to understand that a variety of other factors, such as epigenetics, genetic modifiers, environmental factors, and socioeconomic status, can affect CF outcomes. The long-term management of this complicated multisystem condition has been made easier for patients, their families, and physicians by earlier and more accurate identification techniques, evidence-based research, and centralized expert multidisciplinary care.

## 1. Introduction

The multisystem inflammatory disease known as mucoviscidosis or cystic fibrosis (CF) is linked to a markedly reduced life expectancy, mostly because of the disease’s pulmonary symptoms and associated infections. Approximately 1 in 2500 to 3500 Caucasian and White American births, 1 in 17,000 African American births, and 1 in 31,000 Asian American births are affected with cystic fibrosis (MIM #219700), the most prevalent autosomal recessive fatal genetic illness caused by a defect in a gene on the long arm of Chromosome 7 [1]. This gene codes for the cystic fibrosis transmembrane conductance regulator (CFTR), a chloride channel that is located on epithelial surfaces [1,2]. When the CFTR protein malfunctions, the resulting viscous, inspissated secretions cause blockage, infection, inflammation, and eventually organ destruction. Clinical symptoms of these processes include cirrhosis, intestinal blockage, and obstructive azoospermia from atrophic or nonexistent vasa deferens, exocrine and endocrine pancreatic disorders, and chronic sinopulmonary diseases.

The life expectancy of individuals with cystic fibrosis is constantly fluctuating, but it remains the most prevalent fatal hereditary illness in the Caucasian population. The improvement in anticipated survival in patients with CF has been a real success since the disease was first described as a clinical entity in 1938; however, further major advancements remain to be made [3]. Today, the median life expectancy of patients with CF has risen from a few months in the 1940s to as high as 41 years old, and it is still increasing due to advancements in the genetic, medical, and technological domains; more aggressive use of antibiotics; and rigorous dietary support.

Numerous CF genotypes have been recently identified, many of which are characterized by phenotypically milder alterations. This has led to an increase in the total CF population, which has improved the overall prognosis and outcome statistics. Numerous studies have examined every facet of the illness, found variable risk factors, and biomarkers to track the course of the illness. Better treatment and the development of more phenotypically specific therapies have been made possible by our growing understanding of the disorder [4].

Since *CFTR* mutations were found to be the hereditary cause of CF, scientific research has shifted toward precision medicine, and CFTR modulator medications were first made available in the early 2000s. Potentiators, correctors, stabilizers, read-through agents, and amplifiers are the five main categories of CFTR modulators, which are made to support or restore the function of CFTR. Consequently, the survival rate for CF has significantly improved over the past 10 years, which has implications for patient care and predicting outcomes in this complicated illness. With extended life expectancy, it is vital to better anticipate outcomes and prognosticate in CF; hence, the use of survival or death as an outcome measure has become almost negligible in clinical trials or even in research to predict prognosis. Accurately predicting the prognosis is crucial because each person’s decision to have a lung transplant or to treat specific components of the disease aggressively may be more individualized and suitable, further increasing survival.

The objective of our narrative review is to summarize the most recent information from the specialized literature concerning the value and significance of clinical parameters in predicting outcomes, such as gender, diabetes, liver and pancreatic status, lung function, radiography, bacteriology, and blood and sputum biomarkers of inflammation and disease. We will also analyze how variations in these parameters affect the prognosis of children and people with CF, from the prenatal stage to maturity, with a focus on any associations between genotype and phenotype. After identifying and analyzing these factors, we will discuss their involvement in the therapeutic management of CF patients and identify possible research directions.

## 2. Materials and Methods

Although not systematic, a methodological search was performed throughout the review process to ensure quality, and the manuscript was prepared following the SANRA checklist (Scale for the Assessment of Narrative Review Articles) [5]. The approach involved independent data extraction and quality assessment, which was performed by five of our research team, each contributing to the accuracy and thoroughness of the review.

### 2.1. Search Strategy of Electronic Databases

Between 14 February 2025 and 30 May 2025, we performed a review of the data available with regard to the prognostic factors in the evolution of CF in children and young adults. We evaluated articles from the PubMed provider of academic search engine using the following search terms: (prognostic factors) AND (children) AND (cystic fibrosis OR mucoviscidosis). The search was focused on identifying mainly original studies that explored the prognostic factors and evolution of children with mucoviscidosis, from antenatal period until adulthood, specifically examining any correlations between the genotype and phenotype.

#### 2.1.1. Inclusion and Exclusion Criteria

To ensure the inclusion of the most relevant and high-quality evidence, the studies included in this review met the following criteria: the full text was available online and in English, the study was published in the last 10 years, the methodology was clearly stated, and human subjects were included. Our review included systematic reviews, randomized controlled trials, observational studies, and case studies. This selection provided a broad spectrum of data, ranging from large-scale quantitative analyses to detailed reports of individual patient experiences, to allow for a comprehensive exploration of the relationship between the genotype and phenotype of CF and help to identify prognostic factors. We searched the reference lists of the included studies in order to identify other potentially relevant articles. Duplicate studies, non-relevant studies, and studies examining non-human subjects were excluded.

#### 2.1.2. Selection of Studies and Information Extraction

Using database searches, three authors selected articles for the review. They independently checked each identified study’s abstract and title for eligibility. Consensus was reached if there was dispute among the reviewers; otherwise, a fourth reviewer served as a referee. Non-human research and duplicate references were removed. To find relevant papers that met the inclusion criteria, two other authors analyzed the references of the relevant publications. By adhering to these procedures, we aimed to provide an objective, comprehensive, and up-to-date synthesis of the literature on the prognostic factors and evolution of children, adolescents, and young adults with mucoviscidosis, providing insightful information about this challenging field of pediatric medicine.

## 3. Results

The initial PubMed search yielded a total of 66,293 results for all years and 23,997 from the last 10 years. After applying the filters (full text available online, English language, human subjects, age (birth—18 years; adult—19+ years), and publication types (clinical trials, comparative study, randomized controlled trials, and meta-analyses)), 946 studies remained to be screened for eligibility.

**Figure 1:**
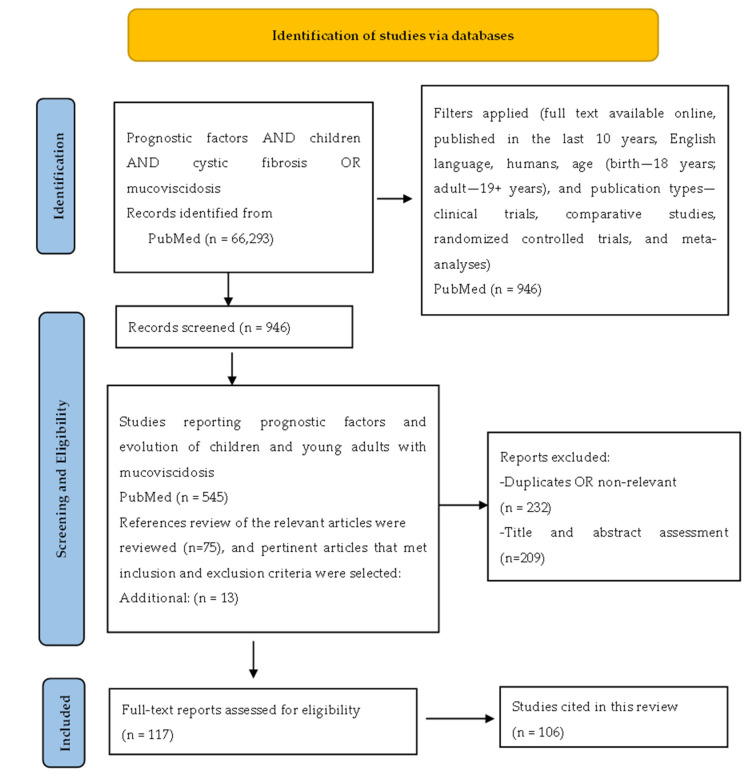
PRISMA flowchart. Notes: PRISMA figure adapted from Liberati et al. [6].

During the second step, we reviewed the titles, abstracts, and full texts of the papers and selected the studies reporting on prognostic factors and the evolution of children and young adults with mucoviscidosis. A total of 545 publications actually reported prognostic factors for CF. We excluded duplicates or non-relevant studies, and after title and abstract assessment, another 209 studies were also excluded; finally, 106 papers were included in our reference list (Figure 1).

## 4. Prenatal Diagnosis of CF and Fetal Risk Factors

More families with inherited disease-causing alleles/variants can be diagnosticated thanks to the increasing use of carrier screening. Prenatal diagnosis has transformed how pregnancies at risk of a hereditary genetic disease are managed. Currently, public health services offer prenatal diagnosis if there is a family member with the disease or if both parents are healthy heterozygous carriers of the same disease. If both parents are carriers, the fetus has a 25% chance of being affected. For couples undergoing in vitro fertilization, pre-implantation genetic diagnosis is now available in some centers.

Chorionic villus or amniotic fluid sampling is used to provide an invasive prenatal diagnosis of monogenic diseases during weeks 11 or 16 of pregnancy. Celocentesis is a novel prenatal diagnostic sample technique that can be used starting at week 7 for couples who may be at risk for genetic disorders [1]. In addition to lowering patient anxiety and offering the choice of medical termination of the affected fetus at 8–10 weeks of gestation—a less traumatic and more secure option than surgical termination in the second trimester—this procedure provides prenatal diagnosis of monogenic diseases at least four weeks earlier than standard procedures. Despite it being limited in sensitivity/scope for CF, evolving, emerging research is now exploring non-invasive prenatal testing (NIPT) for CF. For the 50 most prevalent disease-causing mutations, cell-based NIPT offers a prenatal CF screening plan alternative. A paternal sample is not required for CF screening using circulating trophoblasts, which can be performed via a straightforward, one-visit procedure [7]. As stressed by professional associations, patients must be informed of the advantages and disadvantages of prenatal test options before using cell-free fetal DNA testing and other genetic tests.

Prenatal ultrasound abnormalities such as hyperechoic masses (indicating inspissated meconium in the distal ileum), peritoneal calcifications, polyhydramnios or ascites due to intestinal complications, or non-visualization of the gallbladder, along with an intrauterine growth limitation, may also indicate CF in a high-risk fetus [8]. A common mutation panel or *CFTR* gene sequencing should be used to ascertain the parents’ carrier status if the prenatal ultrasound shows abnormalities. If both parents are carriers of the same disease, adequate genetic counseling is required to address the risks of having a child with CF and its possible repercussions. The delivery should thereafter be planned at a tertiary care center with a multidisciplinary team, and the fetus should be monitored by ultrasonography every six weeks. Premature birth and low birth weight are to be expected; neonates with CF have lower birth weights, mostly because of decreased intrauterine growth, which is only partially explained by shorter gestations [9].

A recent systematic review found no research on potential prenatal management and only three original studies addressing the problem of prenatal ultrasonography results and their implications on postnatal therapy of infants with meconium ileus (MI) and CF [2]. One approach to consider is treating mothers with CFTR correctors and potentiators to work on the fetus’s abnormal CFTR. If started early enough, these medications may change the natural course of MI [10]. Another potential prenatal intervention would be in utero surgical decompression of the obstructed gut or repair of MI to prevent the development of a microcolon, meconium peritonitis, meconium pseudocyst, or intestinal stenosis.

### Prognostic and Clinical Evolution of CF Neonates with Meconium Ileus

Simple or complicated MI (with meconium peritonitis) is typically the initial sign of CF in 12% to 20% of newborns with this genetic disease [11]. Simple and complex MI present differently clinically, ultrasonographically, and radiologically. Simple MI appears from birth as bilious vomiting, a swollen abdomen with dilated intestinal loops evident on the abdominal wall, and the inability to eliminate meconium. Complex MI is characterized by intestinal atresia, prenatal volvulus with gangrene, or perforation of the distended intestinal loops, which can result in meconium cyst and meconium peritonitis. The suspicion of CF will be increased by these clinical signs, and further testing will include sweat testing, thoracoabdominal X-rays, ultrasounds, and genetic studies or biochemical changes in the CFTR, used to confirm the conclusive diagnosis of mucoviscidosis.

Thanks to advancements in surgical techniques, early acceptance of nutritional support for severe MI, and the use of contrast or hyperosmotic enemas as a treatment for simple MI, the prognoses for CF patients with MI are now comparable to those for CF patients without this neonatal manifestation, if appropriate multidisciplinary care is instituted early [2]. Subjects with CF and MI may have lower height and weight percentiles, but their lung function is comparable to that of patients without MI at the ages of 15 and 25. When comparing the nutritional state and pulmonary function of patients with CF and MI to controls who were diagnosed with other symptoms, retrospective investigations found no long-term differences between the groups [2,3,12]. In any case, other studies in this topic indicate that even in this decade, experiencing MI will significantly increase the patient’s morbidity and mortality and place them at a severe disadvantage because CF patients with MI tend to have a higher likelihood of severe CFTR mutations [13]. MI is strongly associated with homozygous severe mutations, especially ***ΔF508*** (F508del/F508del), or G542X, W1282X, and N1303K. Also, infants with MI are much more likely to be homozygous or compound heterozygous for two class I or II mutations and develop severe pancreatic insufficiency [14].

About 50% of individuals with distal intestine obstruction syndrome (DIOS) have previously experienced MI, which is relevant to the long-term development of digestive issues in patients with CF. Fifteen to twenty percent of children and adults with CF have DIOS, which is the MI equivalent [15]. Although a neonate’s presentation with MI did not necessarily predispose them to subsequent development of DIOS, they were more likely to have a more severe form of DIOS that required surgery later in life [16]. Even though MI patients may have a more serious condition, early detection and treatment are beneficial. Patients with CF without MI will receive a later diagnosis; therefore, they have the advantage of having a less severe illness, but they also have the disadvantage of a delayed diagnosis.

## 5. Clinical and Biological Assessment of CF Severity and the Prognostic Implications

Most often, a variety of tests, including birth screening, sweat testing, genetic testing, lung function tests, nasal potential differences, and others, are used to identify CF in children, as well as its severity, in order to predict the outcome.

It is crucial to customize CF patients’ care based on their unique genetics and related comorbidities. The predictive significance of more dynamic clinical condition markers provides more realistic future objectives to center treatment and targets for each patient. These criteria direct practice on a daily basis and categorize patients according to anticipated results.

### 5.1. Cystic Fibrosis Lung Disease

Measurements of pulmonary function have long been utilized as the main indicator of disease severity; forced expiratory volume in one second (FEV_1_) is used to evaluate a patient’s clinical status and predict their death [3]. FEV_1_ measurement is affordable, easily accessible, reproducible, and offers a long-term assessment of airflow restriction over time. An anticipated baseline FEV_1_ of less than 30% or the FEV_1_ decrease rate could precisely identify CF patients who are most at risk of death [17]. The higher incidence of a decrease in FEV_1_ among children and adolescents with good baseline lung function may be explained by the fact that they are less likely to obtain a therapeutic intervention after an acute decline in FEV_1_.

In pediatric CF centers in particular, where lung disease is frequently in its early stages or where there are more technical difficulties in conducting accurate expiratory maneuvers, FEV_1_ does not adequately detect early lung disease when spirometry is frequently normal. Given the remarkable therapeutic discoveries in CF over the past 10 years, particularly in modifying CFTR function, the use of alternative outcomes has become more obvious. This resulted in the development of new imaging modalities such as flurodeoxyglucose positron emission tomography (FDG-PET) imaging and hyperpolarized helium magnetic resonance imaging (He3-MRI), along with greater utilization of multiple-breath washout (MBW) and the Lung Clearance Index (LCI) to determine the severity of the disease [18,19]. Multiple-breath inert gas washout is used to calculate the LCI. There is mounting evidence that this method may be more sensitive than FEV_1_ in identifying early lung disease in preschool children, despite the fact that it is time-consuming and less accessible; future FEV_1_ deterioration may be predicted by aberrant LCI in the presence of normal FEV_1_ [20]. Additionally, the LCI predicts worsening and the time to the first exacerbation, correlates with high-resolution computed tomography (HRCT) and chest magnetic resonance imaging (MRI) results, and may be a useful prognostic index that can more sensitively identify improvements in lung health when evaluating new treatment options [18,21]. Automated quantification techniques are now available to supplement traditional visual studies for a more impartial and repeatable evaluation of illness severity.

Although respiratory failure in CF is a foreseeable consequence of worsening lung disease, there is insufficient data to support its management or clear guidelines for oxygen prescription practices (nocturnal, ambulatory, or resting). Therefore, hypoxia has not been evaluated as a prognostic factor, despite evidence indicating patients at higher mortality risk are associated with poorer six-minute walk test (6 MWT) outcomes, especially in adult subjects with a prediction of FEV_1_ ≤ 60% [22]. This test’s limitations include its inability to identify the mechanism of exercise limitation, the cause of dyspnea or its exacerbation, and the maximum oxygen consumption. Therefore, it is critical to clarify how the 6MWT applies to particular groups, including kids and teenagers with CF. Lung function may be a key predictor of the degree of airway obstruction, but not of functional capacity. This suggests that distinct mechanisms must be at play in the evolution of these variables, as there was no significant correlation found between pulmonary function and walking distance traveled on the 6MWT or work on the 6 min walk test in [23].

#### 5.1.1. Biomarkers of Inflammation in Sputum and Bronchoalveolar Lavage Fluid as a Predictive Factor

Sputum and bronchoalveolar lavage fluid biomarkers may have the potential to serve as outcome measurements in patients with CF. Inflammatory biomarkers TNF-α, IL-8, Neutrophil Elastase (NE), and IL-1β have shown enough validity and responsiveness to be taken into consideration as “pharmacological” measures of medication effectiveness, independent of clinical response [24]. These biomarkers are increasingly integrated into both clinical trials and early disease surveillance programs.

Elevated baseline NE levels in early life were linked to the development of bronchiectasis and a faster drop in percent predicted forced expiratory volume in one second (ppFEV_1_) over the next three years in two pediatric groups [25,26]. Furthermore, free elastase was linked to a decrease in ppFEV_1_ in a larger multicenter trial, showing a −2.9% fall (95% CI: −5.0, −0.9) for every 1-log rise in elastase [27]. Age, chest X-ray results, and time to next pulmonary exacerbation were all substantially correlated with IL-8, while corticosteroid use, microbiota diversity, and evenness were all strongly correlated with NE [24,28]. Because glycosaminoglycan expression is linked to lung neutrophil chemotaxis, its levels may also be a possible biomarker of the course of CF [25].

Sputum biomarkers have potential as a direct indicator of lung inflammation in CF; therefore, a number of additional sputum and bronchoalveolar lavage fluid biomarkers have been investigated, including IL-10 and IL-4, sputum NO_2_/NO_3_, club cell secretory protein, and leukotriene B4; some may become more useful in the future than others. When it comes to identifying children at risk for the onset and advancement of bronchiectasis, elevated levels of IL-8, inducible T-cell costimulator ligand, and hepatocyte growth factor in early children have good sensitivity and fair specificity [29].

In spite of their significance, there are few studies examining biomarker responsiveness to CFTR modulation and anti-inflammatory treatments that predate triple CFTR modulation. Since CFTR modulators might affect potential biomarkers like the fraction of exhaled nitric oxide, metabolomic biomarkers for disease monitoring also need to be reevaluated in the context of their use [30].

It is obvious that large-scale studies are still needed in this regard, as current studies reveal inconsistencies between biomarkers and short- versus long-term response [31]. Also, there are some limitations to biomarker use, specifically in terms of predicting CF evolution: BAL is invasive and less feasible for routine use; sputum quality can vary, especially in young children; and some biomarkers may be elevated in other pulmonary conditions, affecting specificity.

#### 5.1.2. The Effect of Microbial Colonization on CF Prognosis

The primary cause of morbidity and death in patients with CF is progressive obstructive lung disease brought on by a persistent airway infection combined with compromised host immunity. Microbial colonization may reduce the efficacy of CFTR modulators by maintaining a pro-inflammatory environment or by altering mucus properties. The organism’s identification and the ensuing in vitro sensitivity tests aid in directing antibiotic selection, therapy, and even infection management strategies. *Pseudomonas aeruginosa* (PA)*, Staphylococcus aureus* (SA), the *Burkholderia cepacia* complex (BCC), *Achromobacter* species, *Nontuberculous mycobacteria* (NTM), *Haemophilus influenzae*, *Stenotrophomonas maltophilia*, *Aspergillus fumigatus*, and *Candida* species are examples of classical pathogens that are identified in the airways of people with CF.

*Pseudomonas aeruginosa* is the most common bacterial infection in CF patients, especially adults. Early colonization with PA often leads to chronic infection, which is harder to eradicate and accelerates bronchiectasis and respiratory failure. Patients in whom PA colonization is systematically eliminated may see a notable decrease in hospital days and treatment burden; this can decrease the cost of treating exacerbations. However, even with the therapeutic advantages of CFTR modulators, PA clonal lineages continue to exist and could be equally challenging to treat in the future, particularly in patients with advanced lung disease [32]. In patients with *CFTR* G551D mutations, ivacaftor resulted in modest improvements in radiographic pulmonary illnesses and significant decreases in airway inflammation and sputum PA density, and the airway infection caused by PA continued. Therefore, in order to fully benefit from CFTR-targeting medicines, infection control measures could be necessary [31].

*Staphylococcus aureus* is one of the first bacteria to manifest clinically in CF patients. Accurately determining SA’s significance as a pathogen in CF may be challenging due to the intricacy of describing the distinct virulence of the strains in question. When started early in infancy and continued until age six, anti-staphylococcal antibiotic prophylaxis may reduce the number of SA isolates in children, but it is unclear if this result has any clinical significance because antibiotics have side effects, and prolonged use may result in PA infection [33]. Methicillin-Sensitive *Staphylococcus aureus* (MSSA) and Methicillin-Resistant *Staphylococcus aureus* (MRSA) are both acquired early in life, and Hispanic CF patients under 25 years of age are more likely to contract MSSA; therefore, Hispanic people may have higher morbidity due to variations in *S. aureus* acquisition [34]. People with MRSA-positive culture have decreased lung function and need more antibiotics than those with MSSA [30]. Clinical deterioration is often linked to persistent MRSA infection. More than the length of infection, anaerobic growth conditions, which are present in CF airways, influence MRSA’s production of virulence factors and drug susceptibility [35,36].

When comparing PA colonization to MSSA-colonized and non-colonized patients, the former predicts a lower FEV_1_ at the time of culture and a higher rate of decrease in pulmonary function over time. Also, compared to patients infected with either pathogen alone, patients with persistent MRSA and PA co-infection may experience a higher rate of intravenous antibiotic use and deterioration in lung function [37].

One bacterial group that has “natural” multi-antimicrobial resistance is the *Burkholderia cepacia* complex. The BCC can facilitate serious respiratory infections that accelerate lung destruction in individuals with CF. This can be made worse by necrotizing pneumonia with high fevers, leucocytosis, and bacteremia or sepsis, all of which can be fatal; this state is known as “Cepacia Syndrome”. In particular, BCC infection is regarded as a lung transplant exclusion condition [38]. The strains of *B. multivorans* and *B. cenocepacia* have been shown to be the most prevalent and virulent ones. Burkholderia species offer a serious risk to the CF population, yet there is insufficient evidence to support eradication procedures and suggested antibiotic regimens. This is alarming because Burkholderia species may have the worst impact on prognosis of any bacterial colonizer [3]. Standardizing techniques and diagnostic equipment that would enable an early and precise diagnosis is imperative, as is conducting clinical research on the efficacy of currently available antibiotics in curing BCC infections. This will enable us to develop standardized treatment plans for patients infected with BCC.

Infections with *Nontuberculous Mycobacteria* are on the rise worldwide; with an estimated yearly frequency of 12%, pulmonary NTM illness may pose a serious risk to people with CF. From a brief self-resolving infection to NTM pulmonary illness with substantial morbidity, clinical outcomes after NTM acquisition are extremely diverse. Nevertheless, more slow-growing NTM isolates and more post-infection antibiotic use are linked to earlier CF diagnosis, and decline in FEF25-75 is linked to NTM acquisition. Therefore, lung function in CF patients may benefit from early detection and treatment of an NTM infection [39]. A study from 2021 analyzed clinical features, such as age and airway condition at the time of the initial NTM infection, and found comparable data between patients with and without NTM pulmonary illness. *Pseudomonas*, *Streptococcus*, *Veillonella*, *Prevotella*, and *Rothia* were found to have positive correlations with the diagnosis of NTM pulmonary illness and with chronic NTM infection, according to time-series studies of sputum samples taken before the incident NTM infection. Network analysis revealed that participants with and without NTM pulmonary illness, as well as those with persistent versus transient NTM infection, clustered their taxa differently. In conclusion, NTM infection persistence and the diagnosis of NTM lung illness are two outcomes that are linked to the CF airway microbiota before the incident NTM infection. Relationships between NTM results and airway microbiota serve as targets for future treatment and validation as predictive markers [40].

### 5.2. Serum Biomarkers of Inflammation, Nasal Potential Difference, and Sweat Chloride Concentration as Predictive Factors in CF

Serum biomarkers are also attracting interest for use in tracking the course of the disease and forecasting treatment results. For example, serum calprotectin, a protein generated from neutrophils and secreted during neutrophil activation, correlates well with radiological scores, pulmonary symptoms, and the remission of exacerbations. For patients with CF, serum and fecal calprotectin are also tools for tracking inflammatory bowel disorders [41].

Promising options for inclusion in CF anti-inflammatory clinical trials include serum high-sensitivity C-reactive protein and sputum NE, which can reduce variance, prevent redundancy, and function as indicators of the severity and progression of lung disease [42]. The whole-blood leucocyte ribonucleic acid (RNA) level is a more accurate and sensitive biomarker of airway infection than FEV_1_ or C-reactive protein, and it may be even more sensitive when combined with FEV_1_. The effectiveness of inhaled antibiotics and the host’s response to intravenous antibiotic therapy during pulmonary exacerbation episodes are related to the genes of peripheral blood leukocytes that measure inflammation. Subgroups of CF patients with differences in underlying inflammation and varying clinical responses to inhaled antibiotics may be identified via molecular measurement of systemic inflammation [43].

Colonizing microbes trigger chronic neutrophilic inflammation, which damages lung tissue even in the absence of acute infection. *Pseudomonas aeruginosa* infection and pulmonary exacerbations in CF patients have been associated with elevated anti-neutrophil cytoplasmic antibodies specific for bactericidal/permeability-increasing protein levels (BPI-ANCA). Studies in Europe have indicated a substantial correlation between the presence of BPI-ANCA and a poor outcome, such as mortality or the need for a lung transplant, but in the United States, when comparing CF patients who tested positive for BPI-ANCA to those who tested negative, no statistically significant differences were observed in FEV_1_ or the incidence of pulmonary exacerbations [44]. In conclusion, BPI-ANCA’s function in CF patients is still unclear.

The main CF defect, relative CFTR protein activity, is reflected in sweat chloride concentrations. Measuring CFTR function is an important biomarker that provides a valuable indicator of the severity of the disorder and can predict long-term prognosis and the efficacy of treatment. The nasal potential difference and sweat chloride concentration can also be used to measure this. In certain CF genotypes, CFTR modulator therapy can enhance CFTR function and is frequently linked to lower sweat chloride concentrations [28]. Clinical trials using CFTR modulators and correctors, like ivacaftor, have further validated the use of this biomarker—an associated reduction in healthcare resource utilization, as well as improvements in lung function and BMI, is correlated with changes in sweat chloride and nasal potential difference, which are considered indicators of improvement [45].

### 5.3. Genotype’s Impact on the Prognosis of CF Patients

The most well-known of the 2121 possible mutations in CF is delta F508 (c.1521_1523delCTT), which accounts for 66.8% of CF cases (between 26% and 87%, depending on the geographical area) [46]. It causes increased sodium resorption on epithelial surfaces, reduced chloride secretion, and unregulated epithelial sodium channels (ENaCs), and it has long been linked to worse clinical outcomes and more serious conditions. About 17% of the phenotypic heterogeneity can be explained by other known mutations that cause CF symptoms, including G542X, D1152H, W1282X, R553X, G551D, and additional modifier genes [47]. The wide variety of *CFTR* mutations have been divided into six classes (more recently seven classes: class I has been divided into class I and class VII) according to how they ultimately impact a variety of cellular processes, including transcription, intracellular processing, channel location, and the amount of properly functioning protein (Table 1). Classifying the *CFTR* variants based on its impact on quantitative protein synthesis revealed noticeably variations in mortality rates. Higher mortality and a more severe clinical phenotype were observed in groups with significantly lower levels of CFTR (classes I–III) compared to groups with some residual CFTR function (classes IV–VI) [48].

Both mortality and the risk of developing diabetes were influenced by genotype in a retrospective study: adults with severe *CFTR* genotypes had higher mortality at all ages over 32 than those with mild genotypes (*p* = 0.002), and those with severe genotypes also had a significantly higher risk of developing CF-related diabetes (CFRD) (prevalence 60% in adult patients with severe vs. 14% in adults with mild mutations) [49].

In individuals with the class III *CFTR* G551D mutation, ivacaftor is a potentiator medication and targeted therapy that improves gating at the cell surface. Weight gain, decreased pulmonary exacerbation episodes, sustained gains in FEV_1_ of up to 10%, and subjective improvements in quality of life (CFQ-R) ratings have all been linked to ivacaftor administration; however, it does not affect bacterial density or airway inflammation markers [31,50,51].

Along with new animal models and tissue models created from induced pluripotent stem cells, recent developments in gene editing tools will now enable the development of precise gene targeting techniques for the treatment of CF, which could result in successful customized medicines [52]. With the potential for a lasting cure, gene therapy and gene editing hold considerable promise for integration into the treatment of monogenic diseases like CF.

### 5.4. Effect of Gender on Outcome of CF

Multiple studies on prognostic factors of unfavorable outcomes in CF highlight the fact that women are more severely affected than men in terms of pulmonary or digestive involvement, bacterial colonization, the frequency of exacerbations, and survival rate, particularly during adolescence and early adulthood [53,54]. Female gender has also been shown to be an independent substantial risk factor for death, even after controlling for other factors known to affect CF-related mortality. Girls tend to experience a more rapid decline in lung function (FEV_1_), beginning in childhood or adolescence, and the gender gap in lung function becomes apparent early and persists over time, even after HEMT [55].

The explanation for this is that 17β-estradiol (E2), the main female sex hormone, circulates in the body, attached to sex hormone-binding globulin, and interacts with target tissues via a variety of cell-surface-expressed estrogen receptors (ERs). Peak levels of E2 increase the likelihood of infection and eventual aggravation. E2 levels naturally fluctuate during the regular menstrual cycle and further dry the already impaired airway surface fluids found in CF. It has also been demonstrated that elevated E2 increases TLR hyporesponsiveness to a variety of bacteria by inhibiting the release of interleukin-8 (IL-8) [56]. Females often have lower BMI than males with CF, which is another known predictor of poorer outcomes [57].

In comparison to male cohorts, cystic fibrosis-related diabetes prevalence and mortality is also higher at every age in female cohorts [44]. More study is required in this field, especially to better understand the role and possible therapeutic benefits of sex hormones.

Puberty may be delayed in girls, but menarche occurs at the same age or only slightly later for girls with CF compared to their healthy classmates. The estimated rate of subfertility is also higher compared to the general population. Females with CF can often conceive naturally, and with the introduction of CFTR modulator medications, female CF patients may experience an improvement in fertility, with yearly pregnancies rising in tandem [58]. Each woman should individually assess the advantages and disadvantages of modulator therapy during pregnancy and lactation.

Men with cystic fibrosis (CF) have lower fertility, delayed sexual development, and hypogonadism with low testosterone levels, and the risk of their progeny inheriting CF is [59,60]. Infertility is caused by post-testicular obstructive factors; infertile men with cystic fibrosis (CF) are not sterile because they still generate sperm and can use assisted reproductive technology to conceive biological children and have regular, healthy sexual experiences. Before considering fertility treatment, it is recommended that partners be screened for the CFTR gene mutation and that they both undergo genetic counseling.

### 5.5. The Correlation Between CF Prognosis and Exocrine Dysfunction

Pancreatic insufficiency is frequently linked to mutation classes I, II, and III because of a higher level of CFTR malfunction and deficiency. Compound heterozygous people with one less severe allele or mutation from classes IV, V, and VI typically maintain some degree of exocrine function since CFTR is still produced to some degree [61].

Individual genotypes and the range of phenotypic manifestations of exocrine pancreatic dysfunction in CF are tightly related, but over 85% of CF patients experience exocrine pancreatic insufficiency at some stage of their illness, making it a very frequent problem. Before this complication was recognized, affected patients historically perished from malnutrition in the early stages of infancy, before the now-common lung symptoms of CF could appear. Failure to thrive, steatorrhea, and deficits in fat-soluble vitamins are caused by the malabsorption of fat, protein, and micronutrients that results from loss of pancreatic exocrine function; exocrine disease typically appears early in the course of the illness and results from blockage of the pancreatic proximal intralobular ducts by inspissated mucus clogs. A fecal elastase threshold of less than 200 μg/g is typically employed as a marker of pancreatic insufficiency, although a lower cutoff of 100 μg/g has been shown to have a higher predictive value for ruling out pancreatic insufficiency and reduces false positive results due to fecal dilution from non-pancreatic intestinal sources [61]. It has been proposed that the malabsorption blood test could also be used to identify variations in fat absorption between CF patients with and without enzyme administration [62].

A statistically significant drop in FEV_1_% is linked to exocrine pancreatic insufficiency, and patients with this condition are twice as likely to have severe lung disease (defined as FEV < 40% predicted) [63]. This suggests a correlation between a declining prognosis and a lack of pancreatic exocrine function.

Although patients with sufficient pancreatic function often have a less severe phenotype, acute symptomatic pancreatitis can occur in this cohort at a rate of about 10–20%, which typically manifests as recurrent episodes. Pancreatitis should be considered a possible cause for abdominal pain, and the 10–15% of individuals with CF who are born with adequate pancreatic function will require observation for potential future loss of this function.

CFTR modulators effects on pancreatic function may be crucial for enhancing survival and well-being in individuals with CF. It is important to understand which treatments affect the pancreas the most and when this effect should be maximized. Patients with exocrine pancreatic insufficiency require lifelong pancreatic enzyme replacement therapy, nutritional monitoring, and vitamin supplementation, improving weight gain, lung function, and overall survival; the ability to calculate the right dosage and patient adherence techniques are also essential [64].

### 5.6. Cystic Fibrosis-Related Diabetes (CFRD)

As patients age, the prevalence of CF-related diabetes (CFRD) rises dramatically. With an average incidence of 20% in adolescents and 40% to 50% in adults, CFRD is a frequent comorbidity in mucoviscidosis patients [65]. It includes conditions ranging from poor glucose tolerance to CFRD with hyperglycemia during fasting. During pulmonary exacerbations, elevated levels of growth hormone, cortisol, catecholamines, and inflammatory cytokines lead to insulin resistance, which exacerbates insulin deficiency [66]. Glucocorticosteroid use, immunosuppressive treatment after lung transplantation, liver disease, and malabsorption are some of the extrinsic factors contributing to the pathophysiology of the condition, in addition to insulin deficiency or dysfunction brought on by the breakdown or disruption of pancreatic β-cells.

The severity of insulin insufficiency has been linked to the rate of deterioration in FEV_1_% over a 4-year period, indicating that CFRD has a deleterious impact on pulmonary function and a detrimental prognostic impact, with higher mortality linked to worse nutritional status and more severe pulmonary illness [67]. Increased glucose concentrations in the airways resulting from raised blood glucose levels can accelerate exacerbations and contribute to the onset of certain respiratory tract infections. This could suggest that CF patients may see an improvement in their clinical condition if modest changes in glucose metabolism are treated early with insulin secretagogues or short-action insulin [68].

Mild neuropathy is the most prevalent sign of microvascular complications of diabetes mellitus, and its incidence rates are comparable to those in non-CF diabetics. According to a study of 285 CFRD patients aged ≥6 years (out of 775 CF patients) followed for more than ten years, 16% of CFRD patients may also develop retinopathy, and 14% may develop microalbuminemia [69].

Severe *CFTR* variants are more common in CFRD patients. However, in a prior study, death was found to be correlated with both *CFTR* genotype severity and CFRD separately. Risk of CFRD rises notably after age 10–15 and affects >40–50% of adults with CF. The death rate for CFRD patients older than 30 years is still higher than that of CF patients without diabetes, despite significant progress over time [49]. Infections, reduced lung function, and inadequate nutritional conditions are linked to CFRD. With longer life expectancies, early detection and vigorous treatment of CFRD are more crucial than ever [70].

### 5.7. Cystic Fibrosis-Related Liver Disease (CFLD)

CFLD first appears as biochemical abnormalities in liver enzymes. The next stage is structural disease, which presents as elevated bile content in the liver parenchyma or fatty infiltration on ultrasound scans. These disease processes are caused by viscous bile as a result of aberrant CFTR-regulated ion transport across cholangiocytes, which decreases biliary flow and obstructs the intrahepatic bile ducts. This results in bile duct overgrowth, portal tract fibrosis, and inflammatory processes that harm hepatocytes and cholangiocytes [71].

The most clinically significant issue among the several hepatobiliary problems linked to CF is the development of localized biliary cirrhosis into multilobular cirrhosis, which is accompanied by portal hypertension and, possibly, end-stage liver disease. Decompensated liver disease can include ascites, portal hypertension, variceal disease, and coagulopathy. Once substantial portal hypertension has been established, the prognosis is not good. After lung disease and transplant problems, liver illness is frequently mentioned as the third most prevalent cause of death for people with CF, with a 2.5% mortality rate [71,72]. The majority of patients initially present with upper gastrointestinal hemorrhage as a result of esophageal varices and show symptoms of portal hypertension in their second decade of life [73].

With an estimated frequency of up to 30–40%, CFLD is becoming more common as the life expectancy of CF patients increases. Only 5–10% of CF patients develop clinically significant cirrhosis, but this subgroup has markedly worse outcomes [74].

It has been suggested that non-CFTR modifier genes enhance the likelihood of developing CFLD and that detecting these genetic modifiers may help identify individuals at risk early. CFLD and portal hypertension are substantially correlated with polymorphisms in the *SERPINA1* gene, which codes for alpha-1 antitrypsin [75]. Particularly for patients with class I–III mutations, males, those with meconium ileus at birth, and those with related pancreatic insufficiency, surveillance of hepatic symptoms should be concentrated throughout the first ten years of life [73].

For individuals with CF, the function and results of different therapies for controlling advanced liver disease (non-malignant end stage disease) are presently unknown [76]. Currently, there is no proven medical treatment to either treat or stop the course of liver disease. It is still unknown whether CFTR modulators have a direct impact on the long-term results of liver disease, but they do have revolutionary effects on pulmonary, nutritional, and quality of life outcomes. Hepatic side effects linked to drugs can occur often, so it is critical that clinicians understand drug-monitoring guidelines [77]. Ursodeoxycholic acid is often used, though its benefit in altering disease course is debated [78]. Liver transplantation, either by itself or in conjunction with a lung transplant, is a viable option with better results for individuals whose condition is progressing.

### 5.8. Weight and Nutritional Status in Predicting the Course of CF

The effects of malnutrition in CF are particularly intriguing; patients are at a high risk of nutritional deficiencies due to exocrine and endocrine abnormalities, as well as increased basal metabolic needs in the context of chronic illness. It is well established that in CF, low body mass index (BMI) is more common as people age, is strongly linked to declining lung function, and indicates a worse prognosis over a four-year period. Additionally, children and adults with malnutrition were significantly more frequently colonized by PA and fungi and less so by MSSA in [79]. Even in groups with somewhat severe lung illness, boosting the nutritional status of patients with malnutrition may eventually improve their pulmonary function.

In addition to malnutrition, CF patients frequently have low bone mineral density (BMD), which leads to osteopenia and osteoporosis. Lung function and BMD have not yet been demonstrated to be significantly correlated, while age, weight, and BMI are predictors of poor BMD. Oral and intravenous bisphosphonates may improve bone mineral density; however, there is not enough information to say whether treatment lowers fracture rates in affected patients [80]. Patients with CF should have their body composition assessed as part of their regular evaluations.

Recent global trends demonstrate a considerable increase in overweight and obese statuses among people with CF, causing concern over the possible risk of conventional comorbidities linked to age and weight [81]. Numerous bioactive food ingredients, including antioxidants, polyunsaturated fatty acids, and phytochemicals, are beneficial in the treatment of CF and also have a cardiovascular protective effect [82]. Overweight trends in the CF population are accelerated by highly effective CFTR modulator therapy (HEMT), which is linked to increased weight through a number of pathways. HEMT leads to better nutrient absorption (due to improved pancreatic and gastrointestinal function), reduced energy expenditure (less chronic inflammation and lung infection), and improved appetite and well-being. In contrast to the previous general, high-fat, high-calorie dietary advice, in the post-modulator era, CF nutrition guidelines are shifting to more customized methods [83]. In particular, patients with CF who have the F508del mutation have demonstrated an improved nutritional and pulmonary condition when using three-part elexacaftor–tezacaftor–ivacaftor combination treatment [84].

The CF-ABLE score was developed using age, BMI, lung function (i.e., FEV_1_), and the number of exacerbations during the previous three months. The score was verified using information from 370 patients in Ireland’s national Cystic Fibrosis Registry. The ABLE was given a score between 0 and 7. A person receives 3.5 points if their FEV_1_ is less than 52%, 1.5 points if they have experienced more than one exacerbation in the previous three months, and 1 point if their BMI is less than 20.1 kg/m^2^ or their age is less than 24. Within four years, patients with a low score are at very little risk of dying or needing a lung transplant; however, the risk rises sharply as the score rises. Patients with a score of more than five points are 26% more likely to have a negative outcome. This score predicts outcomes more accurately than FEV_1_ alone and is straightforward and applicable [3]. A 2025 study from China confirms that the CF-ABLE and 3-year prognostic scores can be used to predict poor prognosis for CF patients [85].

## 6. Using Radiology to Forecast Prognosis in CF

Techniques for accurately predicting how lung disease will develop in CF patients are crucial for guiding intensive treatment to avoid lung function loss and end-stage respiratory failure. The prognostic and clinical condition correlations of plain radiographs, CT, and MRI differ, and there are several scoring systems to determine the degree of pulmonary involvement. Currently, the most sensitive technique for evaluating the structural alterations in CF is HRCT imaging of the lungs. Since fluorodeoxyglucose positron emission tomography (FDG-PET) imaging and hyperpolarized helium magnetic resonance imaging (He3-MRI) both improve with antibiotic therapy and strongly correlate with abnormalities observed on HRCT, they are both sensitive and practical techniques in the acute context. The cost and availability of these modalities prevent them from being widely used, but as life expectancy rises and the risk of increased cumulative exposure to ionizing radiation from CT and FDG-PET increases, low-dose HRCT or He3-MRI may become more common [86].

Compared to commonly used irradiating techniques, lung ultrasonography (LUS) is an effective diagnostic tool for determining the severity of CF lung disease. When standardization is performed correctly, LUS is quite reproducible [87]. LUS may be used to track consolidation-related exacerbations, determine the length of therapy, and track the development of atelectasis over time or following therapeutic bronchoalveolar lavage. The literature indicates a moderate association between LUS scores and the type, degree, and CT severity score of bronchiectasis when used in follow-up care [88]. The use of LUS to detect pulmonary exacerbations and track the course of the disease may be enhanced by a future validation of ultrasound results specifically in CF patients.

In Table 2 we grouped the main prognostic factors according to evidence strength, modifiability, pre-modulator prognosis impact, or relevance in CFTR modulator era.

## 7. Discussions

CF is a genetic condition affecting several organs and systems, including the pancreas (most affected), which produces digestive enzymes; the colon, which causes malabsorption syndrome; the respiratory system, which includes the upper airways and pulmonary tissue; and the reproductive system, particularly the male vasa deferens, causing infertility.

Patients with CF continue to live longer thanks to advancements in diagnosis, care, and therapy, as well as interdisciplinary collaboration and the establishment of disease-specific centers. Age-related comorbidities are becoming more prevalent as life expectancy rises, particularly when chronic illness is involved. The dynamics and results of the disease are evolving, as are the clinical presentations of the comorbidities that are either directly or indirectly linked to CFTR failure [89,90].

As CF survival and life expectancy increased, it became clear that straightforward and trustworthy prognostic instruments were needed for use in clinical settings. Clinical and radiological prediction tools have become essential resources in the management of CF. They enable us to group patients according to the severity of their condition and, to a certain degree, predict the likelihood of unfavorable outcomes for those with low prognostic scores.

Some key concepts emerged from the body of literature we analyzed.

Public health services offering prenatal diagnosis has revolutionized the management of pregnancies at risk for this genetically transmissible disease. MI is the initial postnatal sign of this illness, and the patient’s life may be in jeopardy due to this type of neonatal occlusion. Although early and late survival rates for about 80% of simple and severe MI cases are now routinely reported [91], there is currently no agreed-upon strategy for diagnostic procedures or treatment approaches [2]. Early recognition and treatment are helpful, even though MI patients may have a more severe form of CF [2].

Since the genotype is frequently the only information about the condition available in early CF diagnosis, it may be helpful as an initial prognostic marker in early CF diagnosis. However, the outcome also depends on nutritional variables, pancreatic insufficiency, pulmonary function, and colonization by PA. We must be careful about linking genotype and phenotype too closely when predicting clinical outcomes and prognosis for CF patients because of the wide phenotypic variance often observed among identical genotypes.

This is primarily evident in the clinical domains of pulmonary function, pancreatic and diabetic conditions, nutritional assessment, and pharmacological responsiveness [71].

Significant advancements have been made in the study of biomarkers of inflammation in CF, including pulmonary and systemic inflammation, which are prognostic of outcome and correlated with clinical condition [24]. This underscores the continuous translational research in this field and provides an increased understanding of pathophysiological alterations in CF. The demand for biomarkers that correlate with other disease markers such as pulmonary function, bacterial colonization, exacerbation frequency, and radiological abnormalities increases as survival in this condition increases.

Sputum biomarkers in CF show promise as indicators of airway inflammation and may offer safety, efficacy, and mechanistic information to evaluate the course of the disease and the effectiveness of treatments [24]. Sputum biomarkers need to be objective, repeatable, and measurable in order to represent physiological or pathological processes and have the potential to be used as outcome measures in clinical studies [92].

While CT scans and biomarkers of airway secretion are the primary indicators of early CF lung illness, FEV_1_ and the rate of FEV_1_ decline continue to be important predictors of mortality in CF patients [85,93]. In both clinical care and research settings, the LCI may also be used to track the development of early CF lung disease and evaluate the impact of treatment [94]. In any case, the LCI is unable to reliably predict HRCT results in children with CF, particularly in babies, based on the data currently available. In school-age children with CF, the change in the LCI reflects a larger percentage of episodes with functional impairment than FEV_1_ [95]. Ultimately, the LCI can be employed as a final metric to evaluate the positive impact of interventions.

The pathophysiologic mechanisms behind each intervention seem to dictate its efficacy as an outcome metric for the effectiveness of medical therapy [96].

It appears that long-term studies are needed to completely elucidate the clinical use of the LCI in both a research context and the day-to-day operations of CF clinics. In the future, comprehensive scores that combine imaging, laboratory, pulmonary function, and clinical data will be crucial for both clinical trial use and ongoing illness prediction.

The use of LUS to detect pulmonary exacerbations and track the course of the disease may be enhanced by future validation of ultrasound results specifically in CF patients. To fully understand the function of LUS in the CF population; however, further investigation is required, especially to clarify its longer-term effects on patient treatment and wider utility [88]. Until then, the lung CF score and LUS are metrics that can be used in tandem to diagnose and track CF lung illness in children [97].

Progressive obstructive lung disease, which is caused by persistent airway infection and weakened host immunity, is a major determinant of disease severity, treatment burden, and life expectancy in CF patients. Long-term colonization encourages biofilm formation and promotes multi-drug resistance, making infections harder to treat; therefore, managing colonization and preventing chronic infection are central to improving outcomes [36,79].

To help with the assessment of illness severity and the prediction of outcomes over a specified period of time, new composite scoring methods that consider numerous features of this multisystem disorder have been created, like radiomics-based CT scores [98]. For example, in a multivariable model, risk factors such as female gender, frequent or productive cough, low BMI (<66th percentile, median in the cohort), at least one pulmonary exacerbation, high FEV_1_ (≥115% predicted, in the top quartile), and airway culture positive for MSSA, MRSA, or Stenotrophomonas maltophilia were linked to a greater subsequent mean annual lung function decline [99].

Since CF treatment is becoming increasingly individualized, it is crucial to be able to precisely track the course of the illness and how effective the treatment is. When combined with ivacaftor, several CFTR modulators enhance efficacy and expand the pool of CFTR variations that can be treated. With the approval of elexecaftor–tezecaftor–ivacaftor in 2019, over 90% of the CF population in the US could now benefit from HEMT. With general improvements in pulmonary health, quality of life, nutritional status, and, in women, enhanced fertility, HEMT has proven to be highly useful. The development of further CF-related comorbidities may be postponed by HEMT [100].

Extrapulmonary symptoms (DIOS, CFRD, and CFLD) and the serious psychological problems linked to chronic illness should not be disregarded as CF survival rates rise. Because depression is linked to negative beliefs about drugs that decrease medication adherence, which may ultimately contribute to a worse prognosis, it is crucial to evaluate individuals holistically when determining their prognosis, looking beyond the medical difficulties of CF. In addition to medical care, psychological treatment can help with anxiety and depression and improve patient quality of life, adaptation, understanding, ability, care decisions, and even medical outcomes because CF is a genetic disorder that damages numerous body systems [101].

Socioeconomic factors that affect survival are also easily disregarded, such as exposure to tobacco smoke, a larger family with more than one person with CF, and lower standards of living and socioeconomic status [101,102,103], all of which predict a negative outcome. Therefore, healthcare spending, socioeconomic factors, and psychological problems play significant roles in the overall health and outcomes of people with CF. Thus, lower socioeconomic status often limits access to specialized CF care, medications (like CFTR modulators), and adequate nutrition [104]. Financial strain, work demands, or a lack of support can reduce adherence to complex daily treatments, and poor housing conditions and exposure to pollutants can worsen respiratory symptoms. For example, children whose parents do not have a college degree are three times more likely to be exposed to smoke, and families with yearly household incomes under USD 50,000 are twice as likely [105]. Public insurance and variable insurance are associated with lower FEV1 and more pulmonary exacerbations at age 6 [106]. Given that discrepancies linked to health insurance may endure into adulthood, it is imperative to confront the specific obstacles that individuals with cystic fibrosis and lower socioeconomic status encounter in attaining their health objectives. It is also imperative to ascertain the origins and reasons for these disparities emerging early in life [107]. Last but not least, chronic illness may disrupt schooling and limit job opportunities, affecting long-term socioeconomic status and health outcomes.

These factors must be considered while evaluating each person with CF’s prognostic indices, even though they are challenging to quantify in composite prediction methods. The natural history of CF has changed significantly with the introduction of HEMT, especially for children who qualify for early intervention. Though they still have clinical significance, traditional prognostic markers including genotype class, pancreatic status, and early microbiological colonization are expected to change in predictive usefulness when early treatment modifies the course of disease. For example, despite high-risk characteristics like MI or early infection, nutritional and pulmonary trajectories may be more favorable, and sweat chloride concentration, which was once a diagnostic sign, is now being used to evaluate therapeutic response. Long-term registry data from the United States, Asia, and Europe will be crucial in validating new risk stratification techniques in the HEMT era, and future prognostic models must include treatment exposure variables.

In the last decade, evidence-based research, centralized expert multidisciplinary treatment, and earlier and more accurate detection methods have helped patients, their families, and doctors manage this complex multisystem disorder.

Study limitations are frequent in non-systematic reviews, and the limitations of this review on CF diagnosis, treatment, and outcome include the large number of studies on various types of impairment within this condition as well as on the treatment and long-term evolution of patients. Retrospective research is common, which restricts how broadly the findings can be applied and raises the possibility of reporting bias. Outcomes and survival rates from a specific country may not be comparable to those from another one due to variations in the two cohorts’ demographics and methods of outcome assessment, as well as variations in treatment. Thus, a particular method of predicting the evolution of a patient with CF may not be valid for all patients with this condition.

Future directions: The risk of irreparable pulmonary and digestive impairment in these patients may be reduced with early CF diagnosis and the initiation of suitable, multimodal treatment. There is an urgent need for future research examining the trajectory and stability of biomarkers in well-defined groups. Focused research must be conducted on biomarkers, and study designs must be based on the physiological significance and specificity of the biomarker to the intended intervention in the population under study. The structure of the multidisciplinary team for CF care, as well as other protocols used to track delayed disease progression, will need to be updated on a regular basis, with a stronger emphasis on the needs of the adult population. Looking towards the future, this review highlights the need to identify personalized risk factors for different groups of CF patients and personalized medicine because what applies to a patient with a severe form of CF identified in the neonatal period may not apply to a patient diagnosed in middle adulthood.

## 8. Conclusions

Over the past ten years, improvements in care, diagnostics, and treatment have impacted the prognosis for CF. Although genotyping offers a way to categorize CF according to direct research and treatment, it is crucial to understand that a variety of other factors, such as epigenetics, genetic modifiers, environmental factors, and socioeconomic status, can affect CF outcomes. Because what is effective for a patient with a severe type of CF diagnosed in infancy may not work for a patient diagnosed in middle adulthood, this review emphasizes the necessity of identifying individualized risk factors for various groups of CF patients in the future. Overall, the long-term management of this complicated multisystem condition has been made easier for patients, their families, and physicians by earlier and more accurate identification techniques, evidence-based research, and centralized expert multidisciplinary care.

## Figures and Tables

**Table 1:** CFTR mutation classes and their impact on cellular processes.

Class	Impact on Cellular Process		Type of Mutation	Pancreatic Status	Variants
I	No functional protein produced	(I) Impaired protein production due to nonsense-mediated decay	Premature stop codons: Nonsense; splicing; deletions	Insufficient	W128X; R553X; G542X; Y122X; 3950delT
		(VII) Absence of full-length mature mRNA			dele2,3(21kb); 1717-1G > A
II	Defective processing and maturation		Missense; small deletions or insertions	Insufficient	F508del; N1303K; G85E
III	Defects in regulation of channel opening (gating defect)		Missense; small deletions or insertions	Insufficient	G551D; G551S; G1349D; S1251N; G178R; G970R
IV	Defective chloride conductance		Missense; small deletions or insertions	Insufficient	R117H; R334W; R347P; G314R
V	Reduction in wild-type mRNA (reduced quantity)		Partial splicing	Sufficient	A455E; 2789 + 5G > A; 3272-26A > G; 3849 + 10kbC > T
VI	Increased turnover of unstable protein at cell surface		Missense; nonsense	Variable	120del23; N287Y; 4279insA; rF508del

**Table 2:** The main CF prognostic factors according to evidence strength, modifiability, pre-modulator prognosis impact, and relevance in CFTR modulator era.

CF Prognostic Factor	Evidence Strength (High, Moderate, Low)	Modifiability (Yes, Partially, Not)	Pre-Modulator Prognosis Impact (High, Moderate, Low)	Relevance in CFTR Modulator Era (High, Moderate, Low)
CFTR Genotype	high	not	high	high
Meconium Ileus	moderate	partially	high	moderate
Pancreatic Insufficiency	high	partially	high	moderate
Early Pseudomonas Infection	moderate	partially	high	moderate
Nutritional Status (BMI)	moderate	yes	high	low
Lung Function (FEV1)	high	partially	high	moderate
Sweat Chloride	low	not	moderate	low
Inflammatory Biomarkers (e.g., IL-8)	high	not	moderate	high

## Abbreviations

BMD: bone mineral density; BMI: body mass index; BPI-ANCA: bactericidal/permeability-increasing protein levels; CF: Cystic fibrosis; CFTR: cystic fibrosis transmembrane conductance regulator; CFLD: Cystic Fibrosis-associated liver disease; CFRD: Cystic Fibrosis-Related Diabetes; DIOS: distal intestine obstruction syndrome; LCI: Lung Clearance Index; LUS: lung ultrasonography; FEV_1_: Forced expiratory volume in one second; HEMT: highly effective modulator therapy; HRCT: high resolution computed tomography; MRI: magnetic resonance imaging; MI: meconium ileus; 6MWT: six-minute walk test; NE: Neutrophil Elastase; PA: *Pseudomonas aeruginosa;* SA: *Staphylococcus aureus;* BCC: *Burkholderia cepacia* complex; NTM: Non-tuberculous *Mycobacteria;* MSSA: *Methicillin Sensitive Staphylococcus Aureus;* MRSA: *Methicillin Resistant Staphylococcus Aureus*.
